# Rapidly Evolved Genes in Three *Reaumuria* Transcriptomes and Potential Roles of Pentatricopeptide Repeat Superfamily Proteins in Endangerment of *R. trigyna*

**DOI:** 10.3390/ijms252011065

**Published:** 2024-10-15

**Authors:** Ruizhen Zhang, Xiaoyun Cui, Pengshan Zhao

**Affiliations:** 1 Key Laboratory of Ecological Safety and Sustainable Development in Arid Lands, Northwest Institute of Eco-Environment and Resources, Chinese Academy of Sciences, Lanzhou 730000, China; zrz18737692101@163.com; 2 Key Laboratory of Stress Physiology and Ecology in Cold and Arid Regions, Gansu Province, Northwest Institute of Eco-Environment and Resources, Chinese Academy of Sciences, Lanzhou 730000, China

**Keywords:** pentatricopeptide repeat superfamily protein, *Reaumuria*, selective pattern, leaf length, adaptation

## Abstract

*Reaumuria* genus (Tamaricaceae) is widely distributed across the desert and semi-desert regions of Northern China, playing a crucial role in the restoration and protection of desert ecosystems. Previous studies mainly focused on the physiological responses to environmental stresses; however, due to the limited availability of genomic information, the underlying mechanism of morphological and ecological differences among the *Reaumuria* species remains poorly understood. In this study, we presented the first catalog of expressed transcripts for *R. kaschgarica*, a sympatric species of xerophyte *R. soongorica*. We further performed the pair-wise transcriptome comparison to determine the conserved and divergent genes among *R. soongorica*, *R. kaschgarica*, and the relict recretohalophyte *R. trigyna*. Annotation of the 600 relatively conserved genes revealed that some common genetic modules are employed by the *Reaumuria* species to confront with salt and drought stresses in arid environment. Among the 250 genes showing strong signs of positive selection, eight pentatricopeptide repeat (*PPR*) superfamily protein genes were specifically identified, including seven *PPR* genes in the *R. soongorica* vs. *R. trigyna* comparison and one *PPR* gene in the *R. kaschgarica* vs. *R. trigyna* comparison, while the cyclin D3 gene was found in the *R. soongorica* vs. *R. trigyna* comparison. These findings suggest that genetic variations in *PPR* genes may affect the fertility system or compromise the extent of organelle RNA editing in *R. trigyna*. The present study provides valuable genomic information for *R. kaschgarica* and preliminarily reveals the conserved genetic bases for the abiotic stress adaptation and interspecific divergent selection in the *Reaumuria* species. The rapidly evolved *PPR* and *cyclin D3* genes provide new insights on the endangerment of *R. trigyna* and the leaf length difference among the *Reaumuria* species.

## 1. Introduction

**Figure 1:**
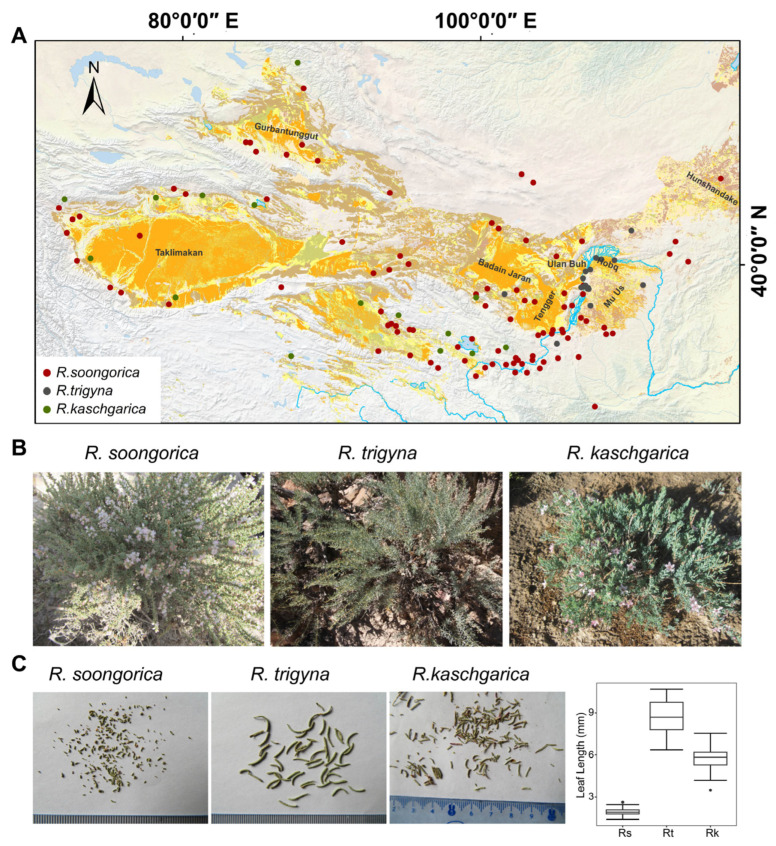
Distribution and morphology of three *Reaumuria* species. (**A**) Distribution of three *Reaumuria* species. The background imagery is from GEBCO_2014 Grid, version 20150318 (http://www.gebco.net), and the desert dataset (in orange) is provided by National Cryosphere Desert Data Center (NCDC, http://www.ncdc.ac.cn/). The map was generated based on the latitude and longitude of the location sites of each species. The information for each red dot of *R. soongorica* was from the natural field survey and previous report [2]. The distribution information of *R. kaschgarica* (green dots) was modified according to the description by Hao et al. [7]. Dark gray dots represent the location of *R. trigyna*, and the information was based on the natural field survey and previous publications [7,8,9]. (**B**) Morphology of *R. soongorica*, *R. trigyna*, and *R. kaschgarica* in their habitat. (**C**) Dry leaves and leaf length of *R. soongorica*, *R. trigyna*, and *R. kaschgarica* (*n* = 20).

*Reaumuria* is a genus adapted to arid environment and wildly distributed across the arid regions of Central Asia [1]. Phylogenetic evidence has revealed that *Reaumuria* is monophyletic, with its estimated divergence from *Tamarix* dating back to the early Paleocene (69.99 million years ago; 66.36 Mya) [1,2]. The perennial xerophytic semi-shrub *R. soongorica* is a constructive and dominant species present in desert ecosystems, which is capable of surviving even after losing up to 95% of its water content [3,4]. Physiological studies have shown that sucrose, malate, and proline are important osmotic regulators and the antioxidative system can effectively remove superoxide anion and scavenge hydrogen peroxide during dehydration in *R. soongorica* [5,6]. Consistent with the findings, a set of genes relevant to aquaporins, proline transporters, flavonoid biosynthetic proteins, small heat shock proteins, and late embryogenesis abundant proteins is highly accumulated after drought stress treatment [3]. *R. kaschgarica* is a sympatric species of *R. soongorica* (Figure 1), and the divergence time between these two species could be dated to middle Miocene (13.13 Mya; [1]). It is characterized by flowers located at the apices of branchlets and longer leaves compared to those of *R. soongorica*.

In contrast with the broad distribution of *R. soongorica* and *R. kaschgarica*, the relict plant *R. trigyna* is restricted to a small region—the Alashan Left and Western Ordos of Inner Mongolia—which is recognized as one of the most noticeable endemic areas of China (Figure 1) [10]. Another characteristic feature of the *Reaumuria* habitat is high soil salinity. Both *R. soongorica* and *R. trigyna* can complete their life cycles in soils with sodium concentrations as high as 0.6 mg/g [8,11]. Thus, salt excretion is considered a crucial mechanism contributing to the salinity of resistance of *Reaumuria* [11,12,13,14]. Transcriptome analyses have revealed significant alterations in the expression levels of genes involved in ion transport and reactive oxygen species scavenging following salt stress treatment in *R. trigyna* [11].

Many specific traits, including succulent leaves, sunken stomata, and salt excretion glands, have evolved in *Reaumuria* species to enable it to survive in an arid environment [11,14,15]. While numerous physiological and molecular studies have elucidated the adaptation mechanisms of *Reaumuria*, the genetic mechanisms underlying the development of specific organs and phenotype diversity are poorly understood for the *Reaumuria* species. Previous study based on neutral markers, RAPD and ISSR, has revealed high genetic variations within *R. trigyna* populations [9]. However, the evolutionary constraints that result in the endangerment of *R. trigyna* remain understudied. In this study, we obtained the transcriptome of *R. kaschgarica* using the Illumina platform and characterized its transcriptome to expand the available information for the comparative transcriptomic analyses of the *Reaumuria* species. We also identified conserved genes and candidate genes, such as *pentatricopeptide repeat* (*PPR*) proteins and *cyclin D3* genes, that have experienced adaptive evolution among *R. soongorica*, *R. kaschgarica*, and *R. trigyna* via comparative evolutionary analyses. PPRs are pivotal in regulating RNA processing within organelles, including the editing, splicing, and stability of mitochondrial and chloroplast transcripts. These processes are crucial for the proper functioning of the photosynthetic and respiratory machinery, which are essential for plant energy metabolism and growth [16,17,18]. Additionally, PPR proteins contribute to the plant’s ability to respond to abiotic stresses [19,20]. Understanding these evolution adaptations not only advances our knowledge of plant evolution but also informs conservation strategies for endangered taxa such as *R. trigyna*.

## 2. Results

### 2.1. Assembly and Annotation of R. kaschgarica Transcriptome

**Figure 2:**
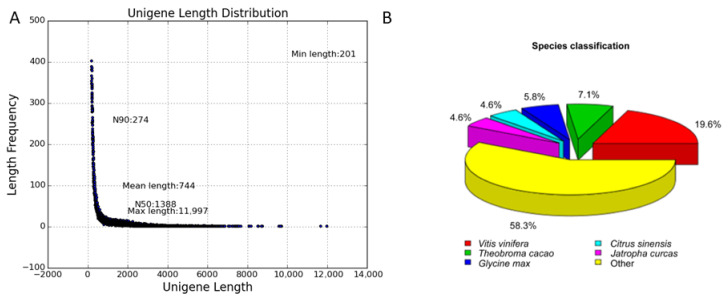
Analysis of the *R. kaschgarica* transcriptome. (**A**) Length distribution of assembled unigenes. (**B**) Taxonomic distribution of the top Blast hits in Nr database for each unigene.

Total RNA was extracted from mixed materials including stems, leaves, and flowers. Paired-end sequencing of the library on a sequencer platform (HiSeq 4000, Illumina, San Diego, CA, USA) yielded 40 million clean reads, with 89.94% of bases having quality scores above Q30. These reads were assembled using Trinity (version r20140413p1) [21], resulting in the generation of 62,680 unigenes with a mean length of 744 bp and an N50 length of 1388 bp (Figure 2A). All unigenes were subsequently aligned against seven public databases, including the NCBI non-redundant nucleotide (Nt) and protein (Nr) database, Pfam, Clusters of Orthologous Groups of proteins (KOG/COG), the Swiss-Prot protein database, the KEGG Ortholog database (KO), and Gene Ontology (GO), using a threshold of less than 1 × 10^−5^. As shown in Table 1, 30,002 unigenes (47.86%) could be matched to protein sequences or domains in at least one of these databases. In addition, the taxonomic distribution based on Nr annotation revealed that 5357 (19.6%) unigenes had top hits to *Vitis vinifera* (Figure 2B). These results were highly consistent with the annotation of *R. soongorica* transcriptome [22].

### 2.2. Characterizing Transcriptomes of R. soongorica, R. kaschgarica, and R. trigyna

**Figure 3:**
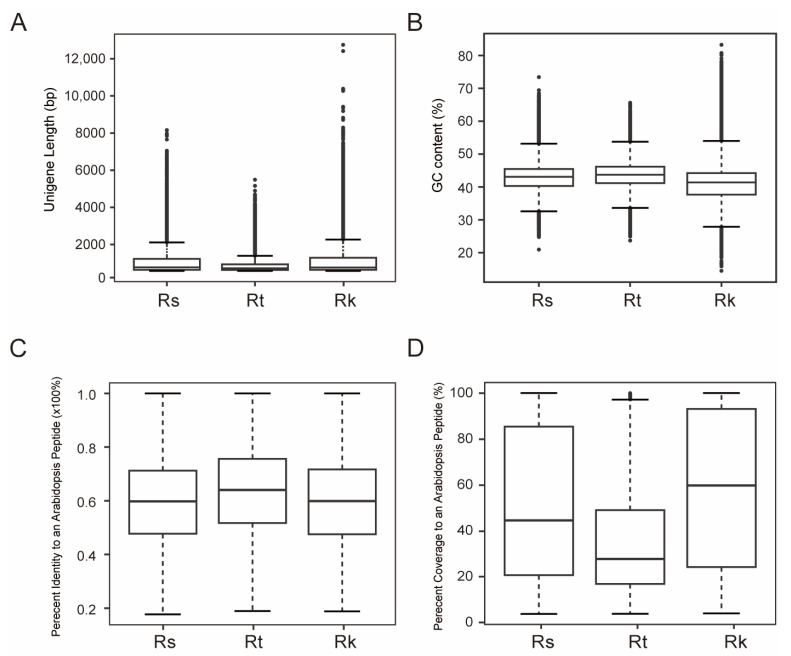
Characteristics of three *Reaumuria* transcriptomes. (**A**,**B**) Boxplots of unigene length and GC content of assembled unigenes. (**C**,**D**) Boxplots of percentage identity and percentage coverage of each *Reaumuria* unigene versus an *Arabidopsis* peptide. The percentage coverage is the longest positive hit/protein length ratio [23]. Rk, *R. kaschgarica*; Rt, *R. trigyna*; Rs, *R. soongorica*.

As a first step towards conducting a comparative evolutionary analysis, we evaluated the transcriptome of *R. kaschgarica* alongside previously published transcriptomes of *R. soongorica* and *R. trigyna* [11,22]. The average length of *R. trigyna* unigenes was 486 bp, which is shorter than those of *R. soongorica* (677 bp) and *R. kaschgarica* (Figure 3A). The GC content of *R. soongorica* (42.3%) and *R. trigyna* (42.9%) was higher than that of *R. kaschgarica* (40.5%; Figure 3B). We further examined the transcriptome quality using a Blastx comparison against the *Arabidopsis* TAIR10 protein database (comprising 35,386 peptides) as described by Dorn et al. (2013) [23]. The percentage of identity and coverage for each unigene versus the most similar *Arabidopsis* peptide were measured independently for three *Reaumuria* transcriptomes (Figure 3C,D). *R. trigyna* exhibited the highest identity value (63.5%) but the lowest coverage (33.9%), likely due to its shorter unigene length (Figure 3A). The high identity values and good coverage suggest that *Reaumuria* transcriptomes are viable genomic resources for further evolutionary analysis.

### 2.3. Comparative Evolutionary Analysis of Reaumuria Transcriptomes

**Figure 4:**
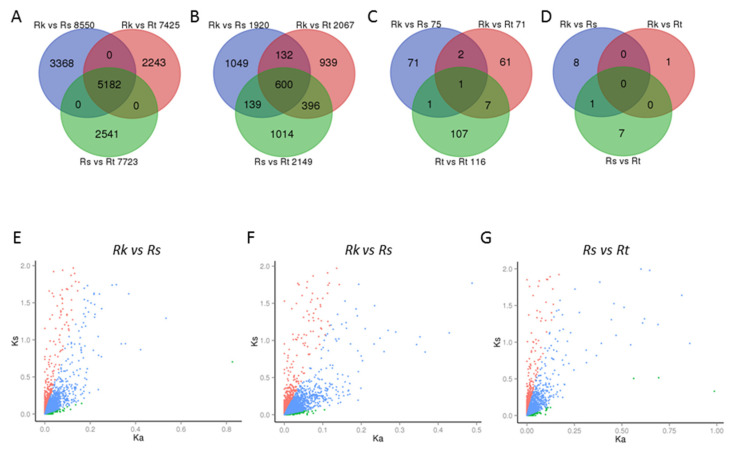
Venn diagram of orthologous genes and scatter diagram of Ka and Ks values for three *Reaumuria* species. (**A**) Orthologous genes of three *Reaumuria* species. There are 5182 orthologous groups, each containing three genes from *R. kaschgarica*, *R. trigyna*, and *R. soongorica*. (**B**) The number of genes under purifying selection in each pair-wise transcriptome comparison. Six hundred relatively conserved genes are identified in three *Reaumuria* species. (**C**) Positively selected genes in three pair-wise comparisons. Only one highly divergent gene is found among three *Reaumuria* species. (**D**) Rapidly evolved PPR genes in *Reaumuria*. (**E**–**G**) Ka and Ks values of three comparisons were estimated. Orthologous genes with Ka > 1 are excluded. Green dots represent divergent ortholog genes with Ka/Ks > 1, red dots indicate conserved ortholog genes with Ka/Ks < 0.1, and blue dots represent no positive or negative selections were detected.

A total of 13,334 orthologous groups were isolated using the OrthoMCL program [24], in which 5182 groups contained three unigenes, each originating from *R. soongorica*, *R. kaschgarica*, and *R. trigyna* transcriptomes (Figure 4A). The non-synonymous (Ka) and synonymous (Ks) nucleotide substitution values were calculated for each orthologous group from the three pair-wise comparisons (Figure 4E–G). The number of orthologous pairs with a Ka/Ks ratio of less than 0.1 was 1920 for *R. kaschgarica* vs. *R. soongorica*, 2067 for *R. kaschgarica* vs. *R. trigyna*, and 2149 for *R. soongorica* vs. *R. trigyna* (Figure 4B). Six hundred orthologous pairs were shared among the three comparisons (Supplementary Table S1), indicating that the sequences of these genes have been highly conserved throughout the evolutionary history of the *Reaumuria* species.

To gain more insights into the functions of highly conserved genes, *Arabidopsis* genes with the highest similarity to each orthologous group were subjected to GO enrichment analysis using the AgriGO program v2.0 [25]. The enriched GO terms were mainly related to basic biology processes, including ‘vesicle-mediated transport’, ‘post-embryonic development’, ‘aromatic compound biosynthetic process’, and ‘protein folding’ (Supplementary Figure S1). Interestingly, the GO term ‘response to radiation’ was also overrepresented, which is consistent with the strong light selection pressure in the habitats of the *Reaumuria* species. Moreover, at least 21 transcription factors, including AP2, bHLH, C2H2, TCP, and NAC, were identified among the conserved *Reaumuria* genes (Supplementary Table S1).

The numbers of orthologous pairs with Ka/Ks ratios above one ranged from 71 to 116 across three comparisons (Figure 4C and Supplementary Table S2). There were eleven groups in which Ka/Ks ratios of one *Reaumuria* gene compared to two other genes were both over one. Only one orthologous group (OG03284), which encoded a heat shock protein (HSP) 20-like chaperone protein, showed rapid evolutionary patterns in all three species. Although none of the GO categories were overrepresented according to the GO enrichment analysis, a number of genes coding pentatricopeptide repeat (PPR) superfamily proteins were identified as positively selected genes (Figure 4D and Table 2). For example, eight PPR genes were detected in the *R. soongorica* vs. *R. trigyna* comparison and one in the *R. kaschgarica* vs. *R. trigyna* comparison.

## 3. Discussion

Physiological studies have demonstrated that the *Reaumuria* species are xerophyte and recretohalophyte plants [4,5,6,8,12,14]. In the present study, 600 relatively conserved genes were identified according to their Ka/Ks ratios (Supplementary Table S1). Highly conserved genes are typically associated with fundamental cellular functions and organismal survival, remaining unchanged throughout evolution because they are crucial for the organism’s survival. For example, the transcriptome analysis of *R. soongorica* and sand rice revealed that the post-embryonic development genes are conserved [26]. The analysis of drought-treated transcriptomes revealed that the expression levels of 18 out of these 600 *R. soongorica* unigenes decreased, while 3 unigenes showed increased expression [3]. Moreover, several ion transporter-related genes, such as *cation*/*H+ exchanger 20* (OG06920), *sodium hydrogen exchanger 3* (*NHX3*; OG08509), and *NHX6* (OG08209), which are involved in the salt response of *R. trigyna* [11], were identified as conserved genes in the *Reaumuria* transcriptomes. The conservativeness of these genes indicates that they are crucial for maintaining ion balance and adapting to high-salinity environments, suggesting that the same genetic modules are employed by three *Reaumuria* species to survive in drought and salinity environments.

Salt excretion is an important characteristic of the *Reaumuria* species [12,13,14]. A recent comparative transcriptome analysis of *Limonium bicolor* has revealed that genes controlling salt gland development may also be involved in trichome formation [27]. Eight genes, such as *GLABRUS 2* (*GL2*), *GL3*, and *TRANSPARENT TESTA GLABRA 1* (*TTG1*), are suggested to be involved in salt gland initiation, and eighteen genes related to plasmodesmata, vesicle transport, cuticular wax, and lignin participate in the ultrastructure differentiation of salt glands [27]. In this study, one orthologous group (OG05769) encoding a TTG1 protein was categorized to be under purifying selection. In addition, the GO term ‘vesicle-mediated transport’ was enriched among the 600 conserved genes. This evidence implies that the mechanism of salt gland development in *Reaumuria* species is similar to that in *L. bicolor* and underscores the key role of vesicle transport in salt secretion [27].

A total of 250 orthologous pairs exhibited rapid evolution with signs of strong selection (Figure 4C), and their orthologous genes in *Arabidopsis* were isolated from the Blastx comparison results to identify the possible functions (Table 2 and Supplementary Table S2). The diversification of these genes may be driven by environmental pressures or ecological interactions, thereby enhancing the organism’s ability to adapt to specific ecosystems. For example, the *HSP20*-like chaperone showed rapid evolution in all three comparisons and the corresponding *Arabidopsis* protein targets to the matrix of peroxisome, which is an important organ to scavenge the reactive oxygen species (ROS) [28]. The *RING E3 ubiquitin ligase ATL78* (homologoue of OG13726), which exhibited Ka/Ks ratios greater than one in the *R. kaschgarica* vs. *R. trigyna* and *R. soongorica* vs. *R. trigyna* comparisons, plays a role in the ROS-mediated abscisic acid (ABA) signaling pathway, conferring drought tolerance [29,30]. Therefore, the variation in these genes suggests that different *Reaumuria* species may have developed unique adaptations to varying local environmental conditions, such as drought stress.

The PPR family is known as one of the largest gene families in terrestrial plants, with *Arabidopsis* alone containing around 450 members. The expansion of this family is likely attributed to the retrotransposition events [31,32]. PPR proteins are sequence-specific RNA-binding proteins with universal influence on the RNA processing within organelles [31]. Organelle RNA metabolism significantly influences plant photosynthesis, respiration, and environmental responses [16,17,18,19,20]. Large variations in the RNA editing extent and products have been reported among different *Arabidopsis* ecotypes, which can be explained by the ecotype-specific variations in PPR protein sequences [33,34,35,36,37]. Previous molecular evolution studies have shown that some *PPR* genes are maintained under strong negative selection, whereas the tandem repeated genes, such as the restorer of fertility (*Rf*) and Rf-like (*RFL*) genes, are subjected to positive selection in *Arabidopsis* [32,33,38]. In this study, we focused on potential functions of the 8 rapidly evolved *PPR* genes that were specifically identified in *R. soongorica* vs. *R. trigyna* or *R. kaschgarica* vs. *R. trigyna* comparisons (Figure 4D). One gene of interest, *RFL9* (homologue of OG14530), acts as a mitochondria RNA editor limited to *Arabidopsis* accessions genetically close to Columbia-0 and is associated with the suppression of a cytoplasmic male sterility in *Arabidopsis* (CMS; [33]). It is possible that some variations fixed in *R. trigyna RFL9* gene could affect the fertility system leading to the endangerment of *R. trigyna*. PPR proteins are also involved in other biological processes, and mutations of *PPRs* can result in diverse phenotypes, such as aberrant leaf development, slow growth, embryo abortion, and hypersensitivity to environmental stresses or ABA [31,39]. Among the rapidly evolved *PPR* genes, *BIGYIN/FISSION1A* (homologue of OG10059) encodes a protein that is triple-targeted to peroxisomes, mitochondria, and chloroplasts, and the disruption of its expression leads to growth inhibition and abnormal fission of peroxisomes and mitochondria in *Arabidopsis* [40,41,42,43]. Numerous studies have demonstrated that both biotic and abiotic stresses can alter the expression patterns of PPR genes. Research has identified various PPR genes that are linked to responses to salt stress, drought stress, cold stress, and defense mechanisms [44,45]. For instance, Luo et al. (2022) conducted an analysis of PPR gene expression in rice under different stress conditions [46]. Their findings revealed that out of the total PPR genes studied, 16 out of 81, 15 out of 127, and 27 out of 35 were either upregulated or downregulated in reaction to osmotic, salt, and oxidative stress, respectively [46]. For the *Arabidopsis* homologs of the remaining six orthologous groups, various expression patterns have been shown in *Arabidopsis* seedlings subjected to different abiotic stresses (Supplementary Figure S2; [47,48,49]). The possibility that variations in these genes could compromise the adaptation ability of *R. trigyna* to the arid regions cannot be ruled out.

Leaf length difference is one of significant phenotype variations among *R. soongorica*, *R. trigyna*, and *R. kaschgarica* (Figure 1), and we are still far from knowing how this variation happened during the evolution of the *Reaumuria* species. Cyclin D3 is known to play an important role in the control of cell number in developing leaves in *Arabidopsis* [50]. For example, the overexpression of *AtCYCD3;1* resulted in significant alterations in leaf architecture, including the loss of distinct spongy and palisade mesophyll layers and the presence of an epidermis made up of numerous poorly differentiated polygonal cells [51]. Furthermore, the overexpression of *AtCYCD3;2* led to the formation of rosettes resembling propellers, featuring narrow, dome-shaped leaves [52]. The finding that *cyclin D3* (OG19460) has been under positive selection in the *R. soongorica* vs. *R. trigyna* comparison implies a possible reason for the shorter leaves observed in *R. soongorica*. Moreover, there were eight rapidly evolved *PPR* genes in the *R. kaschgarica* vs. *R. soongorica* comparison. Mitochondrial editing factor 29 (homologue of OG14044) dually targets mitochondria and chloroplasts in *Arabidopsis*, and mutation of its ortholog *PPR2263* in maize results in narrow and short leaves [53]. However, the *R. trigyna* unigene was absent in this orthologous group (OG14044), indicating that further experiments are necessary to elucidate the role of this gene in leaf development.

Overall, these genetic differences could potentially contribute to *R. trigyna*’s endangerment by affecting its fertility, growth, stress tolerance, and overall adaptation to its environment. This means that the species’ endangerment could be exacerbated if these genetic factors negatively impact its ability to thrive and reproduce in its natural habitat. However, a further study is needed to confirm the roles of the identified PPR proteins and cyclin D3 genes in stress tolerance and reproductive success. This can be achieved through techniques such as gene editing and expression analysis. Additionally, it is also necessary to understand how these genetic variations interplay with environmental factors like drought and salinity. Comparative genomic analyses with other arid-adapted species and population genetics studies of *R. trigyna* will further elucidate the mechanisms underlying its adaptability and inform conservation strategies.

## 4. Materials and Methods

### 4.1. Plant Materials, RNA Extraction, Transcriptome Sequencing, and Analyses

Fresh shoots of *R. kaschgarica*, including stems, leaves, and flowers, were sampled in Dulan County, Qinghai Province, China. The average annual precipitation of Dulan County is 179.1 mm. The distribution areas of *R. kaschgarica* have an average altitude of 2613.9 m (ranging from 1050 to 4931 m), an average annual temperature of 2.4 °C (ranging from −7.8 to 13.2 °C), and an average annual precipitation of 192.5 mm (with a range from 27 to 545 mm). Solar radiation varies from 0.17 to 0.78 kJ m^−2^ day^−1^, with an average of 0.43 kJ m^−2^ day^−1^. The wind speed ranges from 13,751 to 16,953 m s^−1^, with an average of 15,888 m s^−1^. Water vapor pressure varies from 1.3 to 4.1 kPa, with an average of 2.7 kPa (Supplementary Figure S3). All materials were immediately frozen in liquid nitrogen and brought back to our laboratory for analysis. The stems, leaves, and flowers were pooled, and total RNA was extracted using a Plant Total RNA Kit (#DP432, TIANGEN, Beijing, China). The RNA purity was measured using spectrophotometric A260/A280 and A260/A230 ratios, ensuring that the ratios were greater than 2. RNA integrity was assessed using agarose gel electrophoresis and automated systems, such as the Agilent Bioanalyzer 2100 (Agilent Technologies, Santa Clara, CA, USA), ensuring a RIN value above 7 and checking for DNA contamination. To create the library, 25 µL of RNA at a concentration of 470 µg/µL was used. The cDNA library was constructed following the description by Zhao et al. [54] and sequenced using the Illumina Hiseq 4000 platform and 150 bp paired-end reads at Novogene (Beijing, China). A total of 6 Gbp of clean bases were generated, with 89.94% of the bases having a quality score of Q30 or higher.

Clean reads were assembled using Trinity (r20140413p1; [21]) with default parameters. The assembled unigenes were annotated by aligning using Blast against several public databases, including Nr, Nt, Pfam, KOG/COG, Swiss-Prot, KO, and GO, using an e-value cutoff of 1 × 10^−5^. Gene frequency units can be expressed as FPKM (number of Fragments Per Kilobase of transcript sequence per Millions base pairs sequenced), which normalizes for gene length and sequencing depth.

### 4.2. Transcriptome Characterization Using Blastx Program Versus Arabidopsis Protein Database

The *Arabidopsis* protein database (containing 35,386 peptides) was downloaded from the TAIR10 release (www.arabidopsis.org, accessed on 17 October 2015). Unigenes from *R. kaschgarica* in this study, as well as those from *R. soongorica* [22] and *R. trigyna* [11], were compared with the *Arabidopsis* peptides using the Blastx program (v2.2.18), employing a threshold of less than 1 × 10^−5^. The best *Arabidopsis* hit for each unigene was isolated based on sequence identity and e-value [26]. Statistical analyses were conducted using the R software (v4.4.1), as described by Dorn et al. [23].

### 4.3. Ortholog Grouping and Ka/Ks Calculation

The *Reaumuria* orthologous genes were identified using the OrthoMCL method [24]. To analyze the evolutionary pressures on these genes, we calculated the non-synonymous (Ka) and synonymous (Ks) nucleotide substitutions in three transcriptome comparisons (*R. kaschgarica* vs. *R. soongorica*, *R. kaschgarica* vs. *R. trigyna*, and *R. soongorica* vs. *R. trigyna*) using the Codeml program of Phylogenetic Analysis by Maximum Likelihood (PAML) package (version 4.9a) with the basic model [55]. By comparing the ratio of Ka to Ks, we can infer the type of selection pressure acting on the gene. If Ka/Ks > 1, it indicates positive selection; if Ka/Ks = 1, it suggests neutral evolution; and if Ka/Ks < 1, it implies purifying or negative selection. In this study, genes with 3 > Ka/Ks ratios > 1 were considered to be under positive selection pressures, indicating adaptive changes in their sequences [56], whereas genes with Ka/Ks ratios < 0.1 were selected as under purifying selection, which acts to maintain the function of genes [57].

### 4.4. GO Enrichment Analysis

The orthologous *Arabidopsis* genes for each *Reaumuria* group were subjected to GO annotation using the AgriGO program with all parameters set as default [25]. Enriched GO terms were analyzed using the hypergeometric test and corrected by Yekutieli (FDR under dependency). The cutoff of FDR was set at 0.01, with 46 biological processes, 59 cellular components, and 21 molecular function-related terms enriched for the 600 relatively conserved *Reaumuria* genes.

## 5. Conclusions

The first catalog of expressed transcripts of *R. kaschgarica* was presented, and the pair-wise comparisons of *R. soongorica*, *R. trigyna*, and *R. kaschgarica* identified 600 relatively conserved genes and 250 rapidly evolved genes among the *Reaumuria* species. Gene annotation suggests that the same genetic elements underlie the salt and drought stress adaptations of the *Reaumuria* in extreme habitats, with some equivalent components being potentially involved in salt gland development. The driving forces behind the endangerment of *R. trigyna* remain uncertain. The study of the rapidly evolved *PPR* genes provides novel insights on this evolutionary issue. Genetic variations in *PPR* genes could result in abnormal fertility systems or compromise the organelle RNA editing extent of *R. trigyna*. Experimental analysis of the rapidly evolved genes provided in this study will enhance our understanding of the morphology and evolution of the *Reaumuria* species.

## Figures and Tables

**Table 1:** Summary of the annotation of *R. kaschgarica* assembled unigenes.

	Number of Unigenes	Percentage (%)
Nr	27,325	43.59
Nt	13,142	20.96
KO	9330	14.88
Swiss-Prot	19,696	31.42
Pfam	18,959	30.24
GO	19,414	30.97
KOG	10,199	16.27
Annotated in all databases	4131	6.59
Annotated in at least one database	30,002	47.86
Total Unigenes	62,680	100

**Table 2:** Partial list of genes with positive selection. Note: n.d. means no data available.

Orthologous Groups	Genes	Gene Symbols	Ka/Ks		
			Rk vs. Rs	Rk vs. Rt	Rs vs. Rt
OG03284	Rk|c19117_g1 Rs|Unigene52639_A_Rs Rt|Unigene3477_Rt	*HSP20*-like	1.52	1.329	1.20
OG14217	Rk|c41245_g1 Rs|Unigene22663_A_Rs Rt|Unigene66872_Rt	*PPR*	1.02	0.69	1.20
OG13726	Rk|c17221_g1 Rs|CL3395.Contig2_A_Rs Rt|Unigene50964_Rt	*ATL78*	0.97	2.09	1.07
OG10059	Rs|Unigene46659_A_Rs Rk|c6241_g1 Rt|Unigene62804_Rt	*BIGYIN/FIS1A*	0.22	0.0001	1.69
OG14530	Rs|CL10303.Contig1_A_Rs, Rt|Unigene64640_Rt	*RFL9*	n.d.	n.d.	1.62

## Data Availability

Raw data are available in the NCBI Sequence Read Archive (SRA) under accession number PRJNA1138088.

## Supplementary Materials

The supporting information can be downloaded at: https://www.mdpi.com/article/10.3390/ijms252011065/s1.

## Abbreviations

Rk	*R. kaschgarica*
Rt	*R. trigyna*
Rs	*R. soongorica*
PPR	pentatricopeptide repeat
HSP	heat shock protein
Ka	non-synonymous nucleotide substitution
Ks	synonymous nucleotide substitution
NHX3	sodium hydrogen exchanger 3
TTG1	transparent testa glabra 1
ROS	reactive oxygen species
RFL9	restorer of fertility like 9
CMS	cytoplasmic male sterility
