# Supplementary material for: Rapidly Evolved Genes in Three Reaumuria Transcriptomes and Potential Roles of Pentatricopeptide Repeat Superfamily Proteins in Endangerment of R. trigyna
Source: Int J Mol Sci. 2024 Oct 15;25(20):11065. doi: 10.3390/ijms252011065 (PMC11508020; doi:10.3390/ijms252011065)

## Slide 1
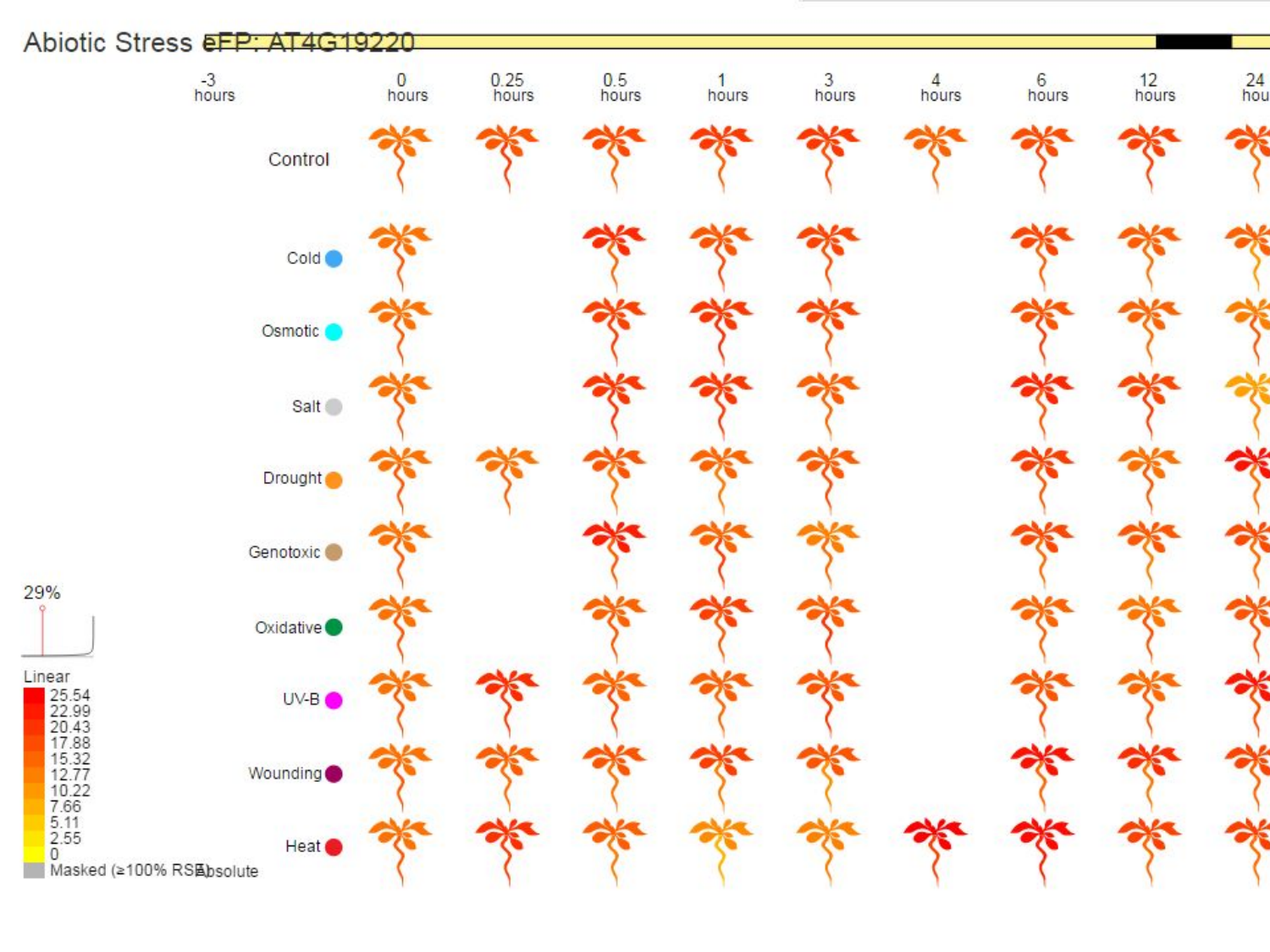

## Slide 2
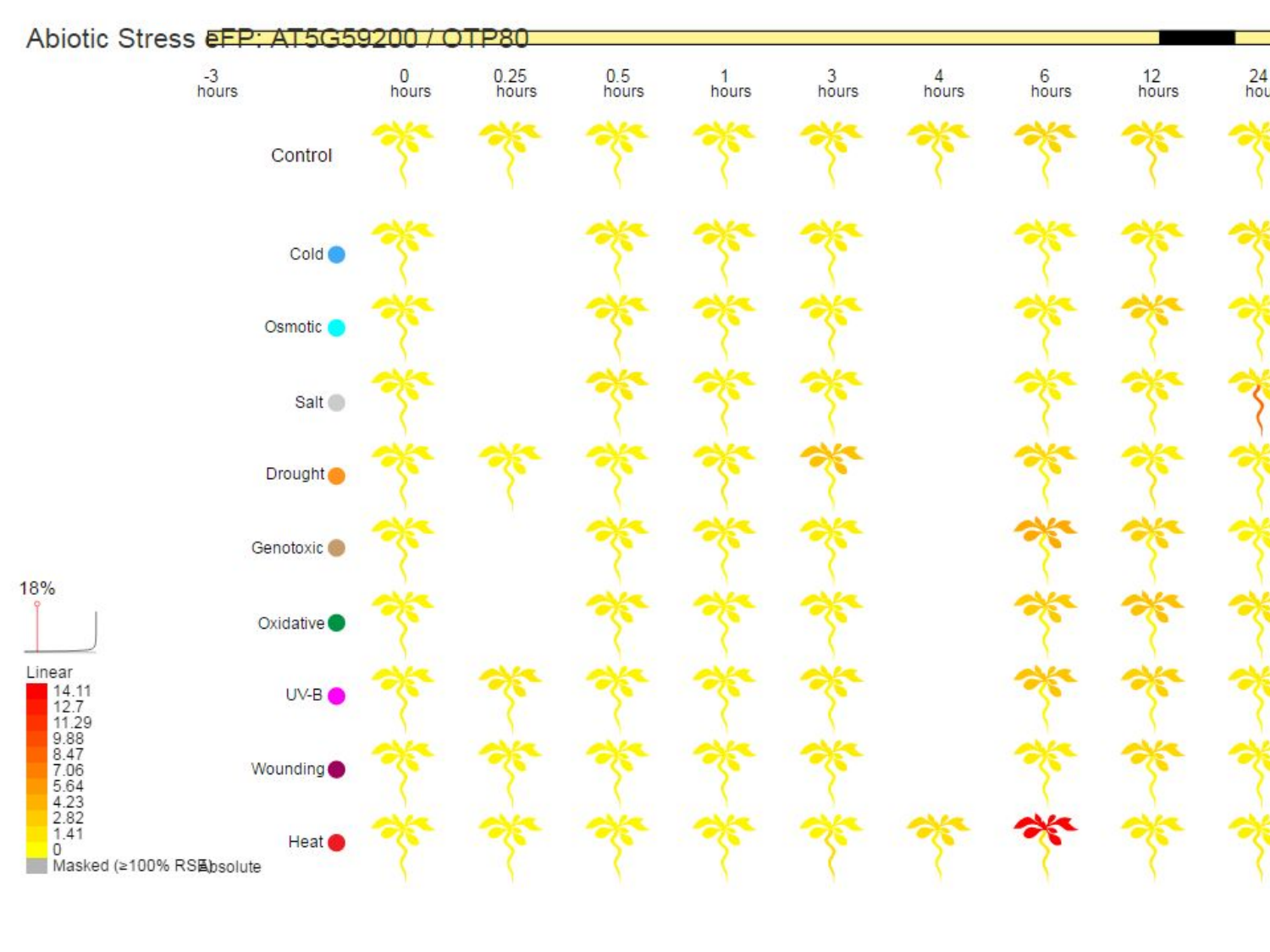

## Slide 3
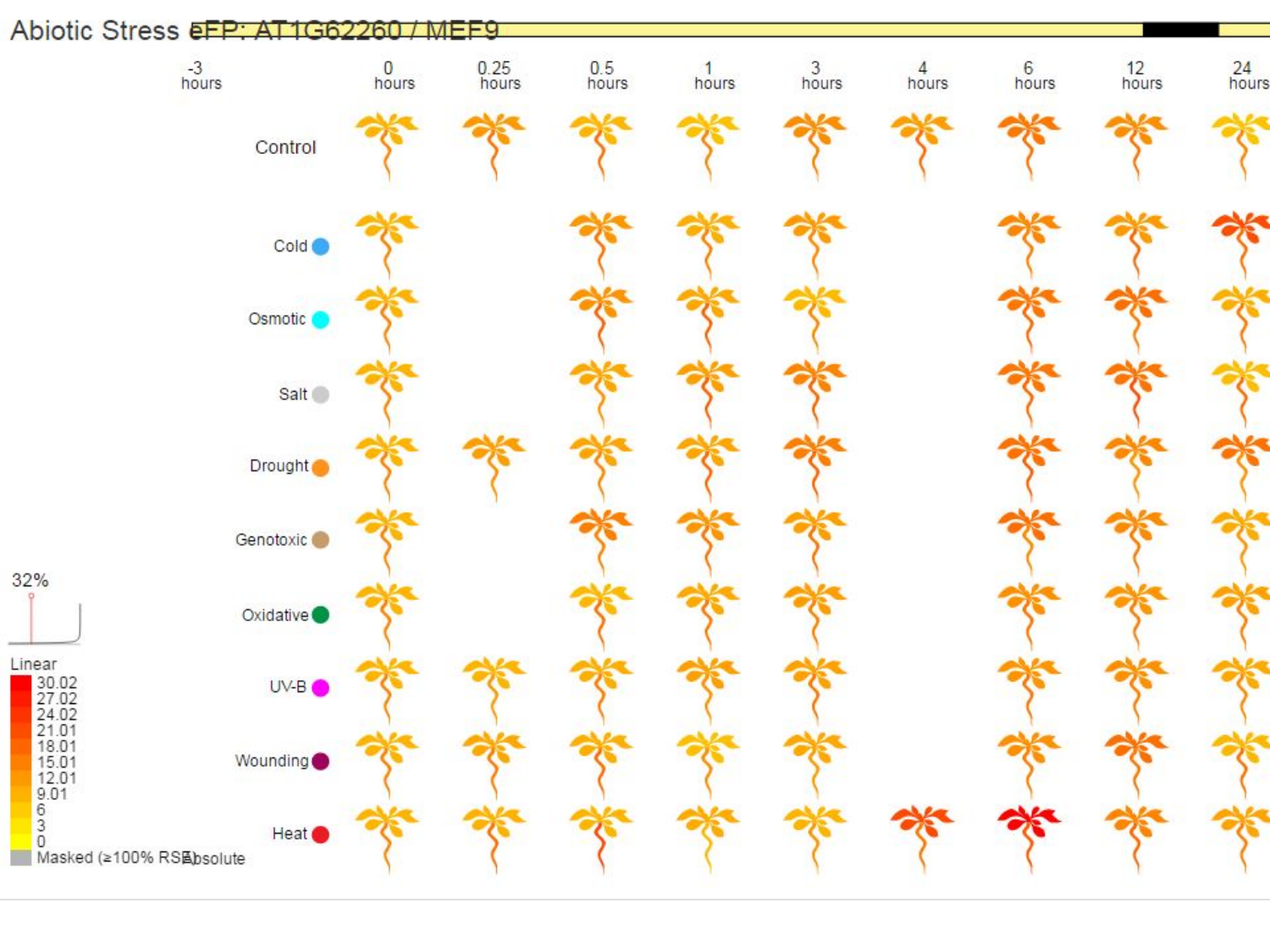

## Slide 4
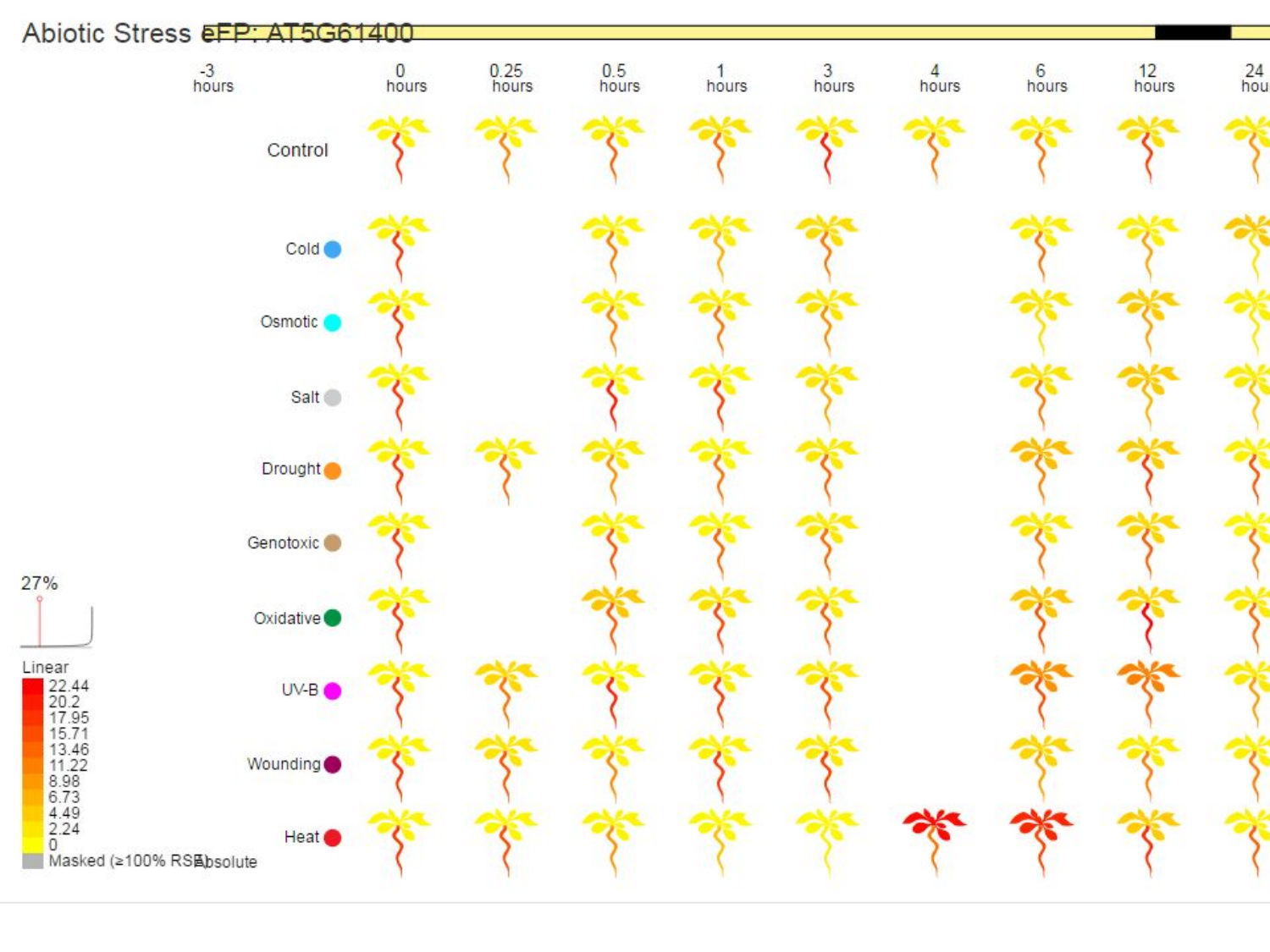

## Slide 5
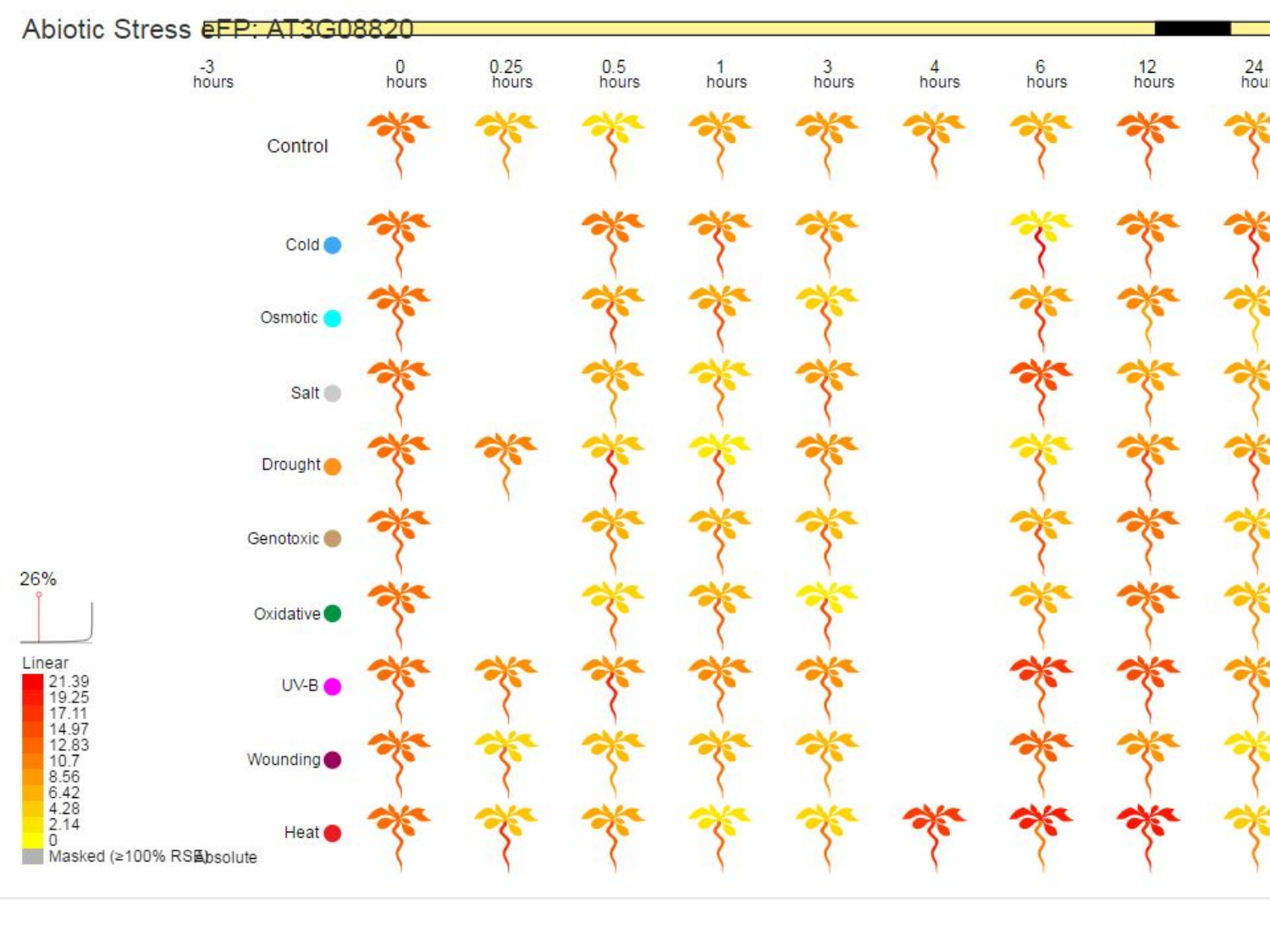

## Slide 6
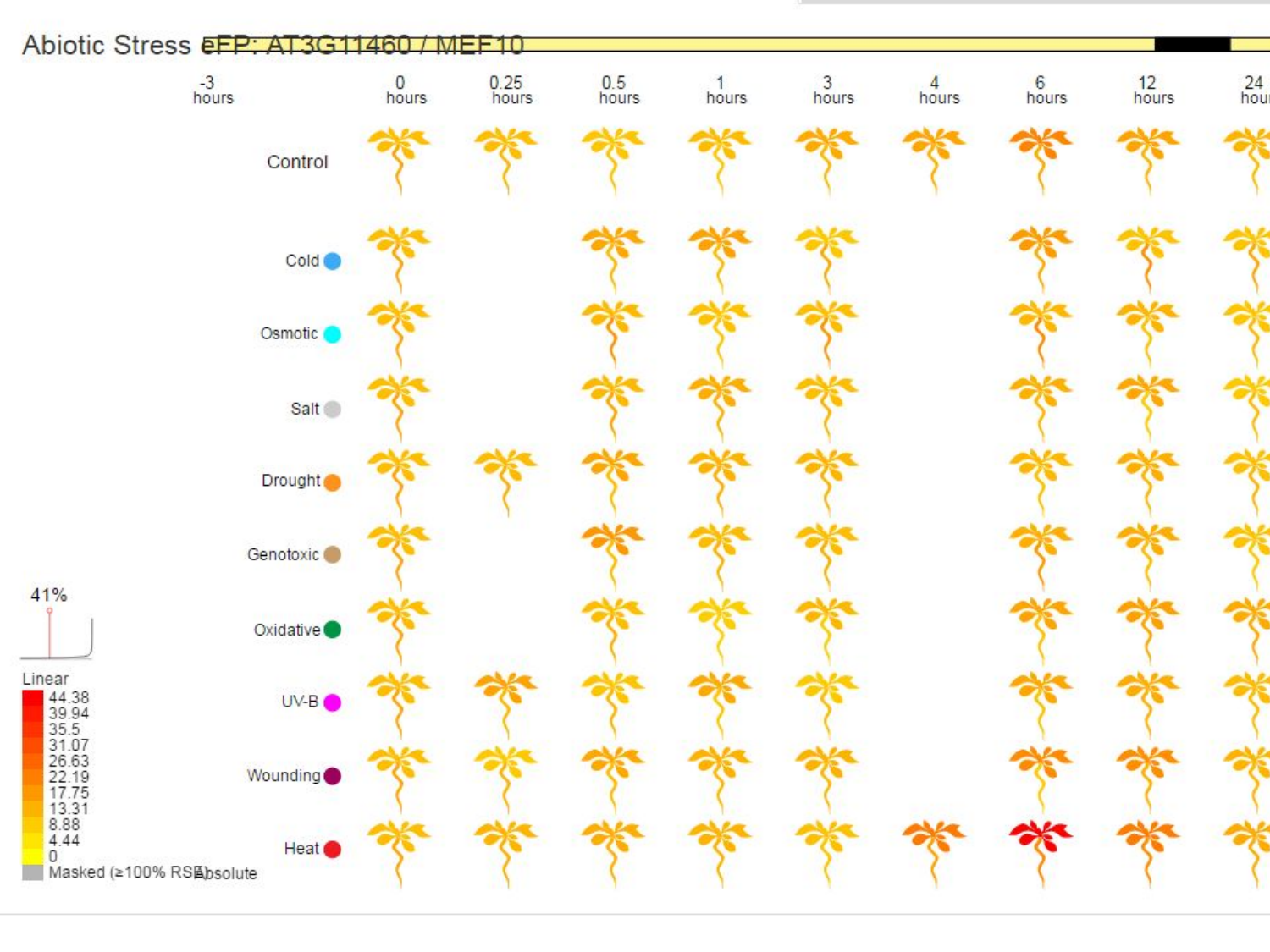

## Slide 7
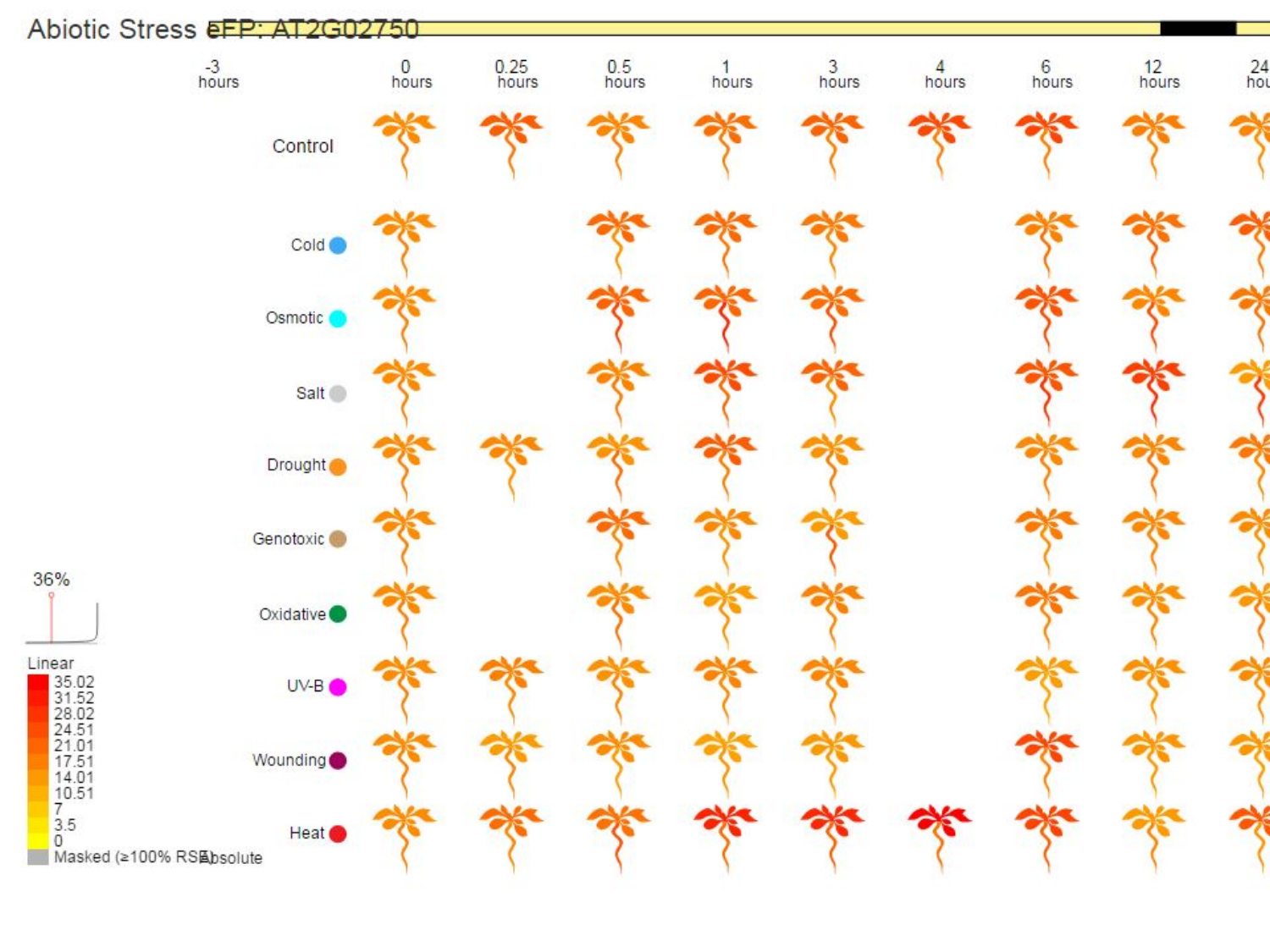

## Slide 8
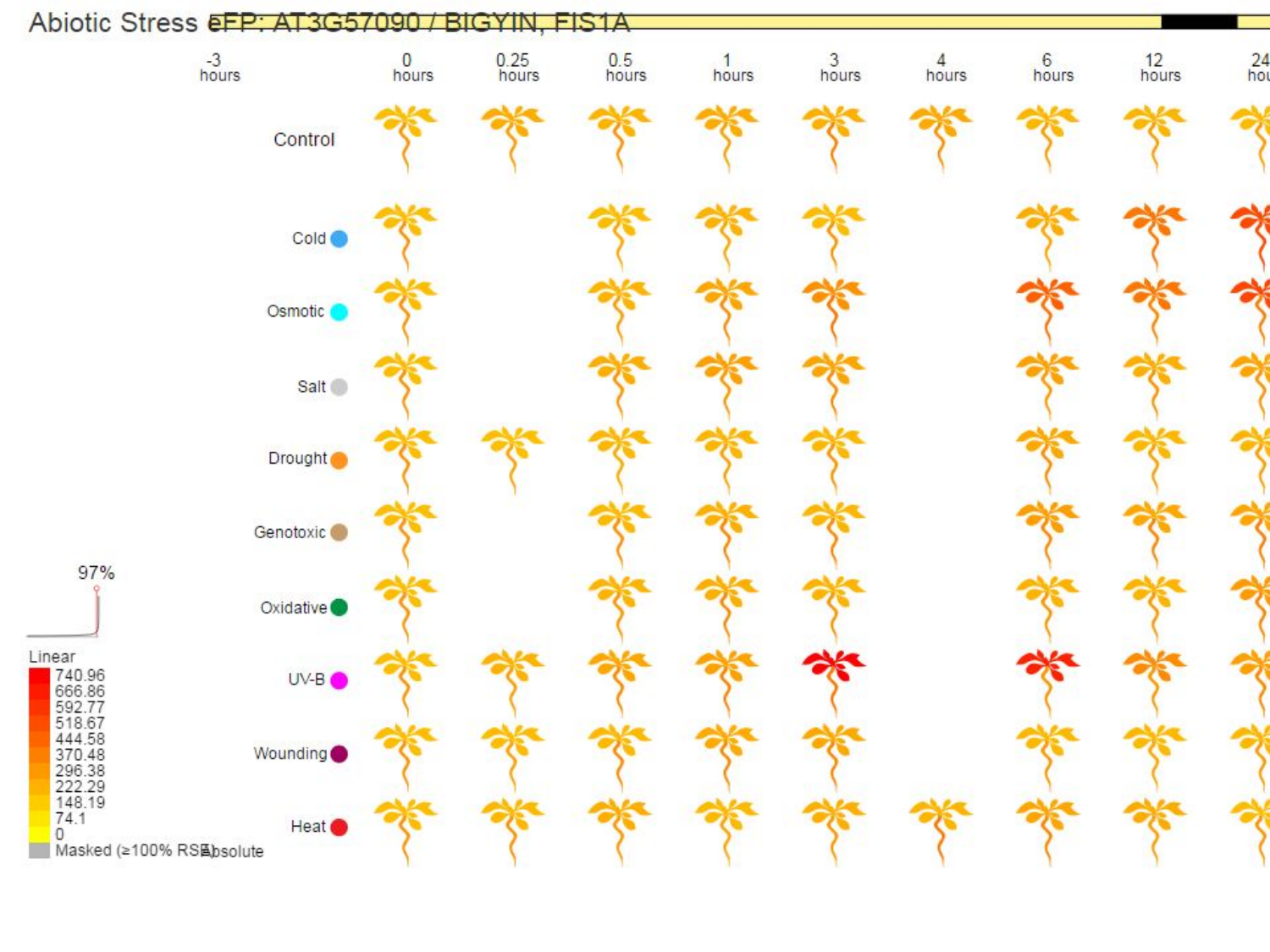

## Slide 9
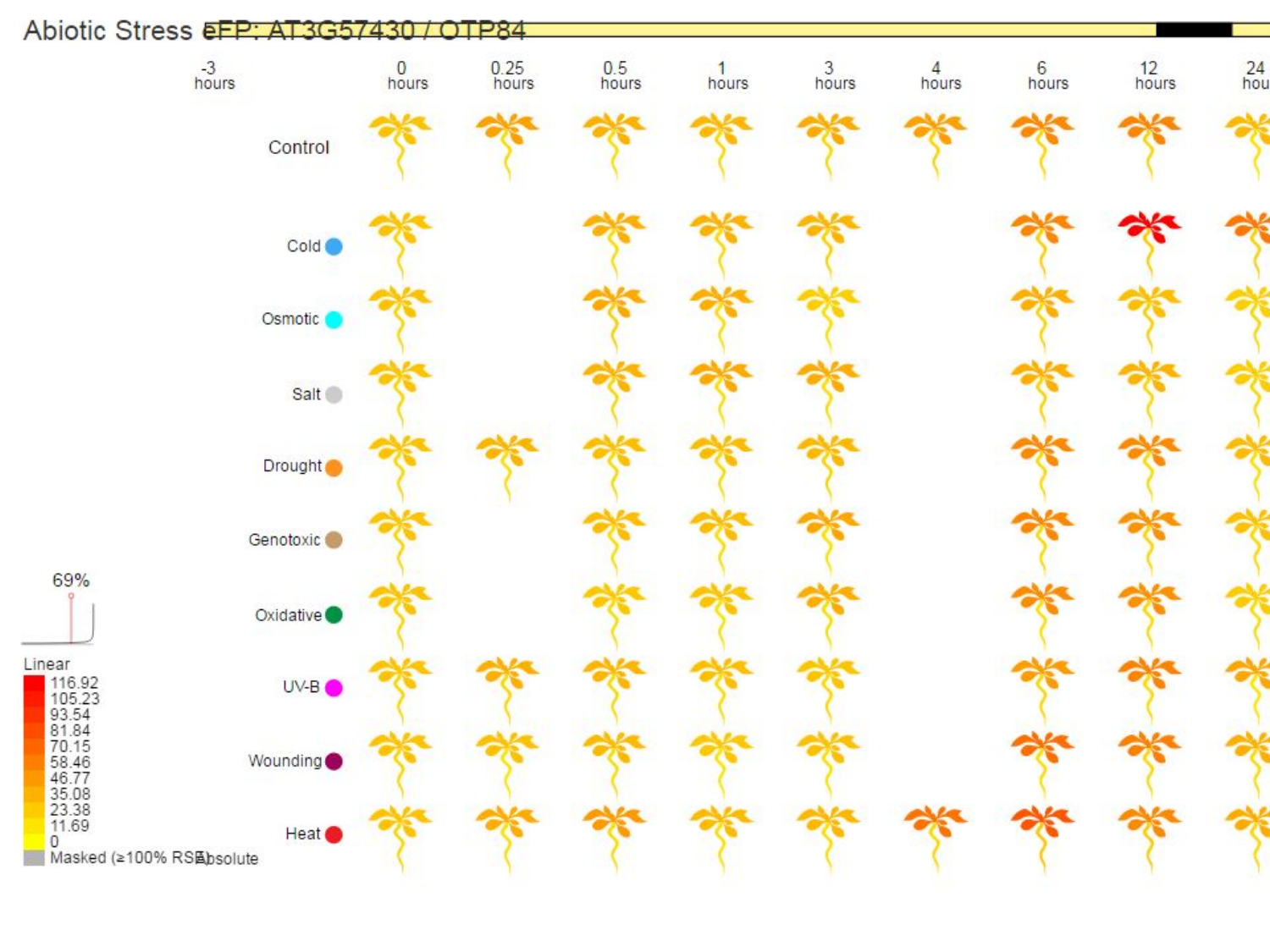

## Slide 10
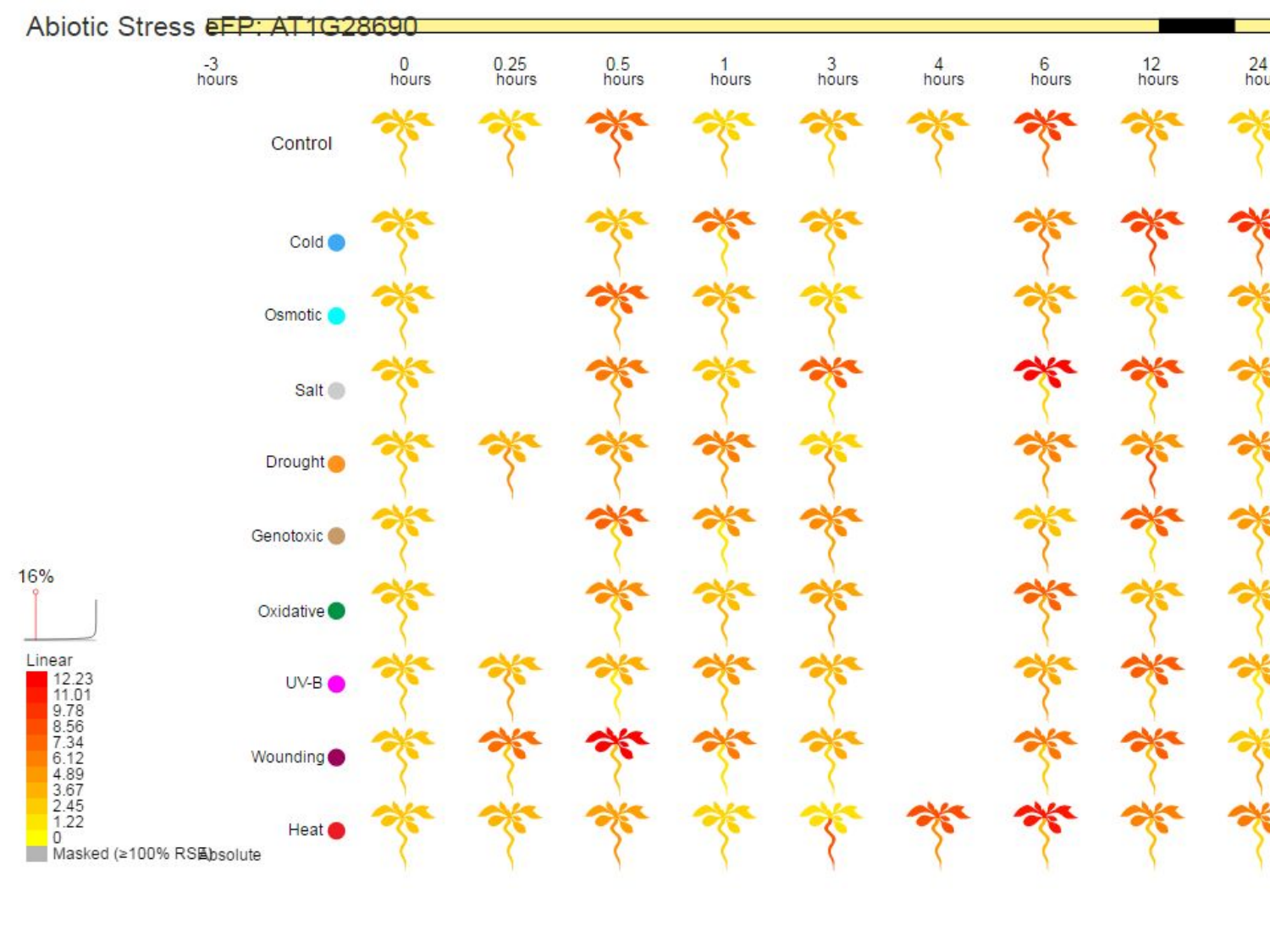

## Slide 11
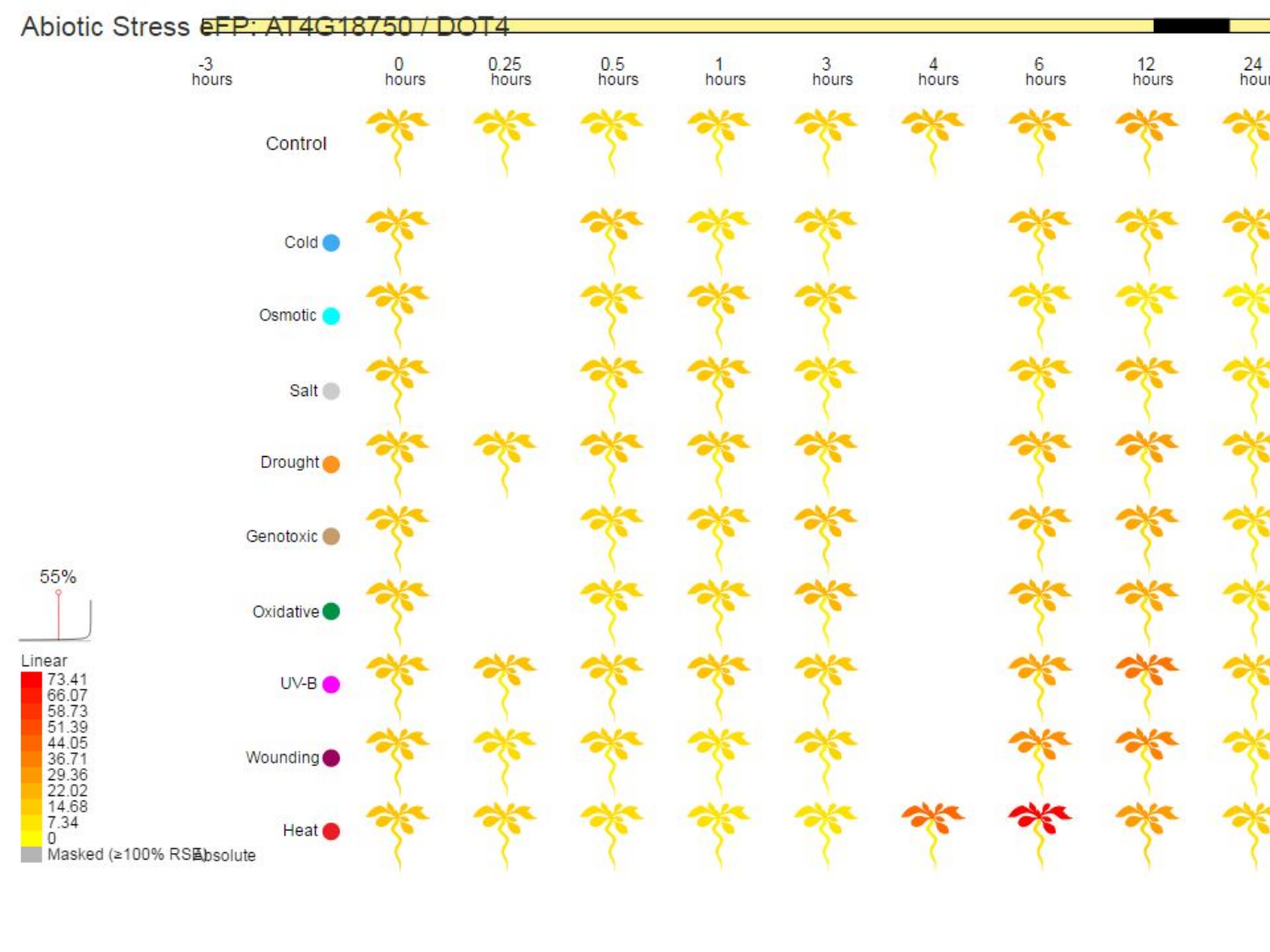

## Slide 12
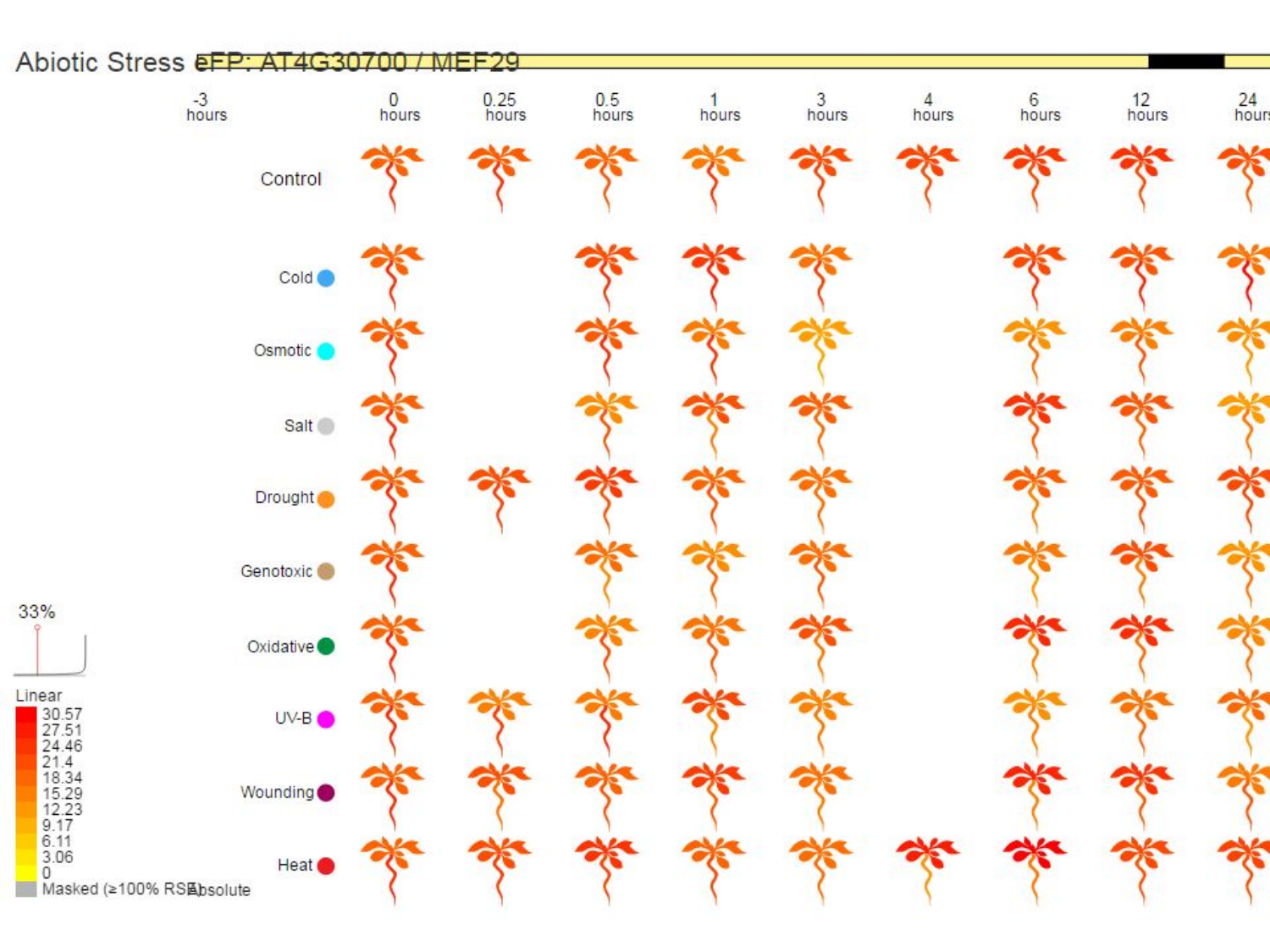

## Slide 13
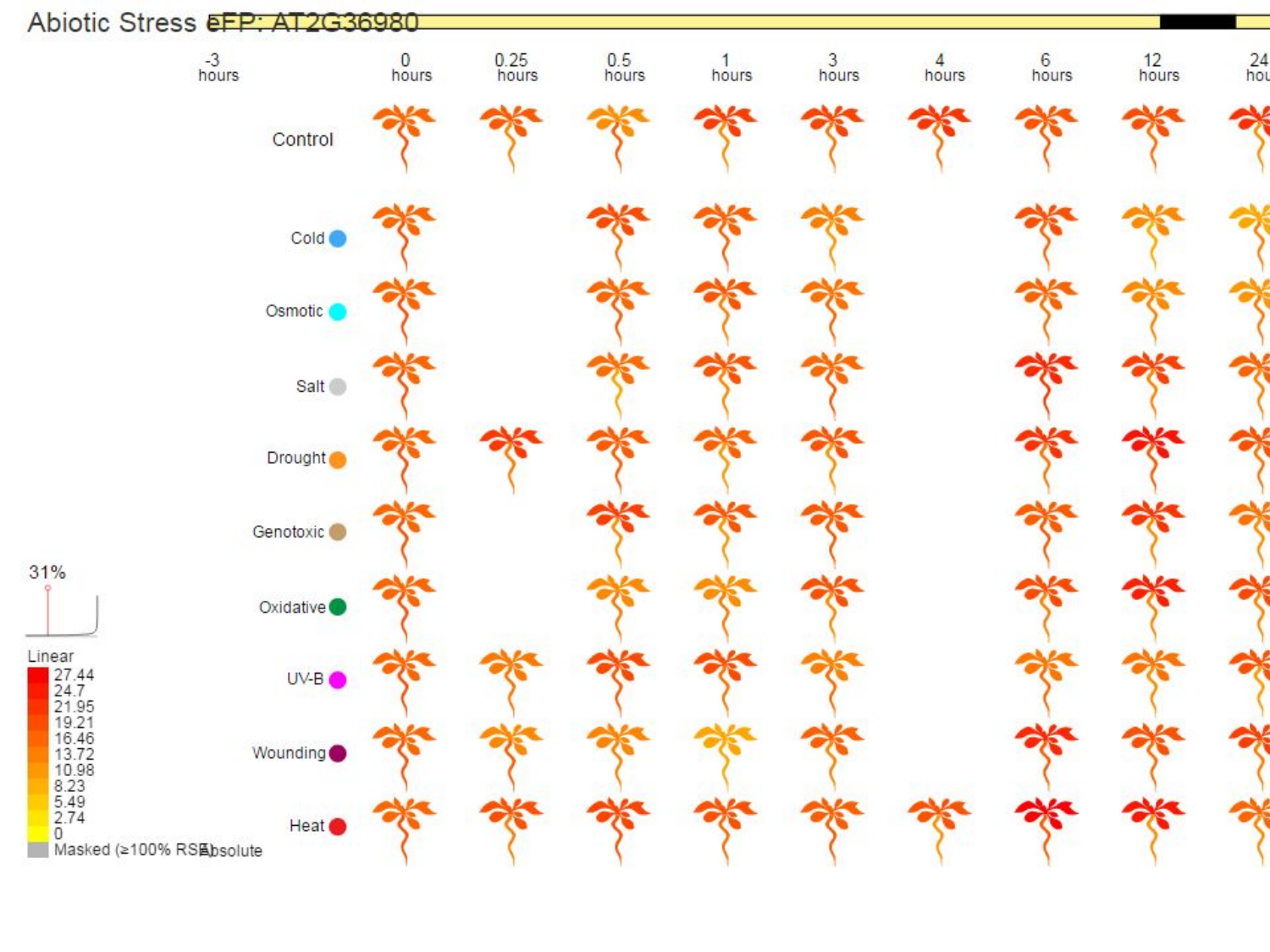

## Slide 14
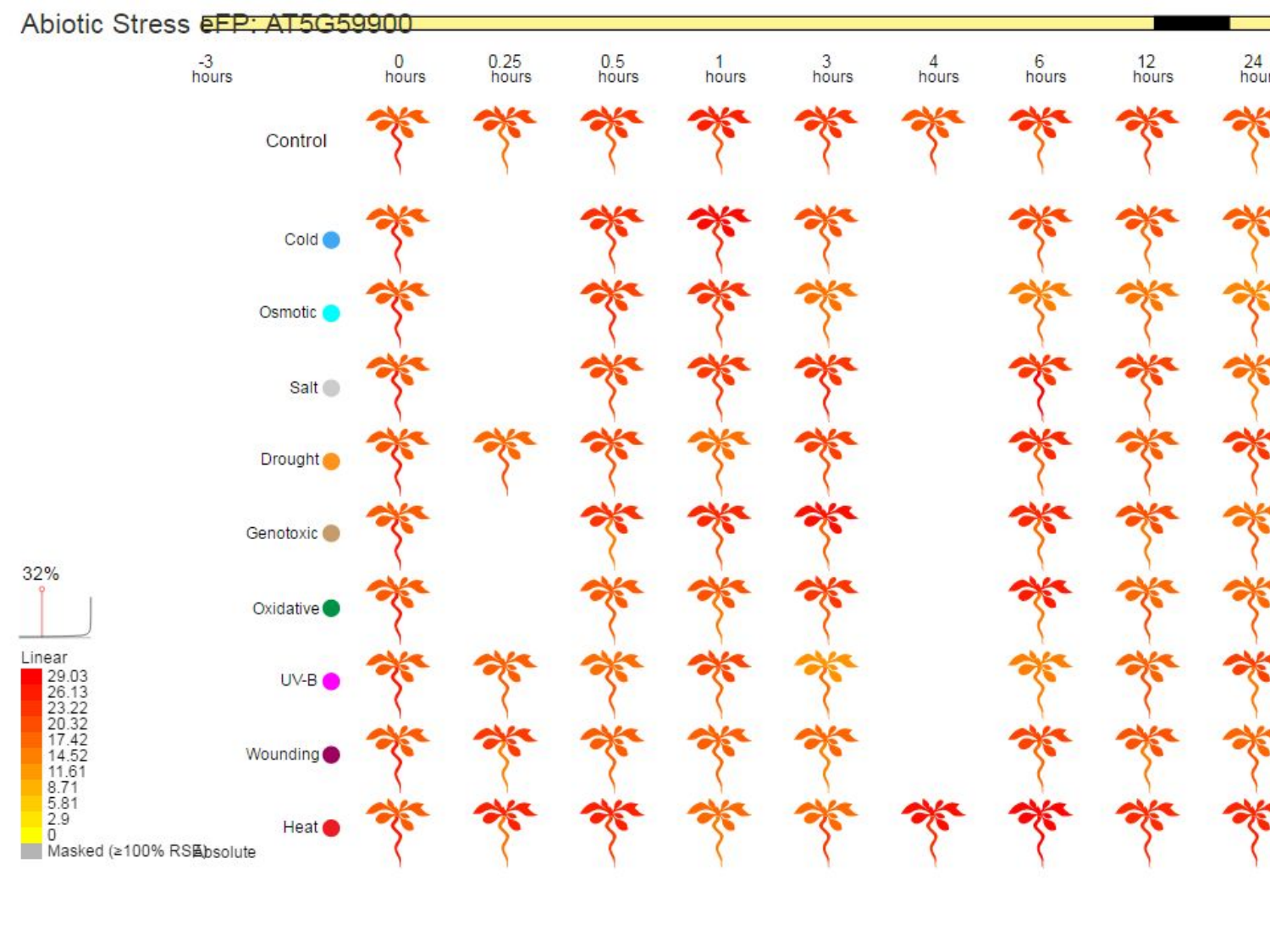

## Slide 15
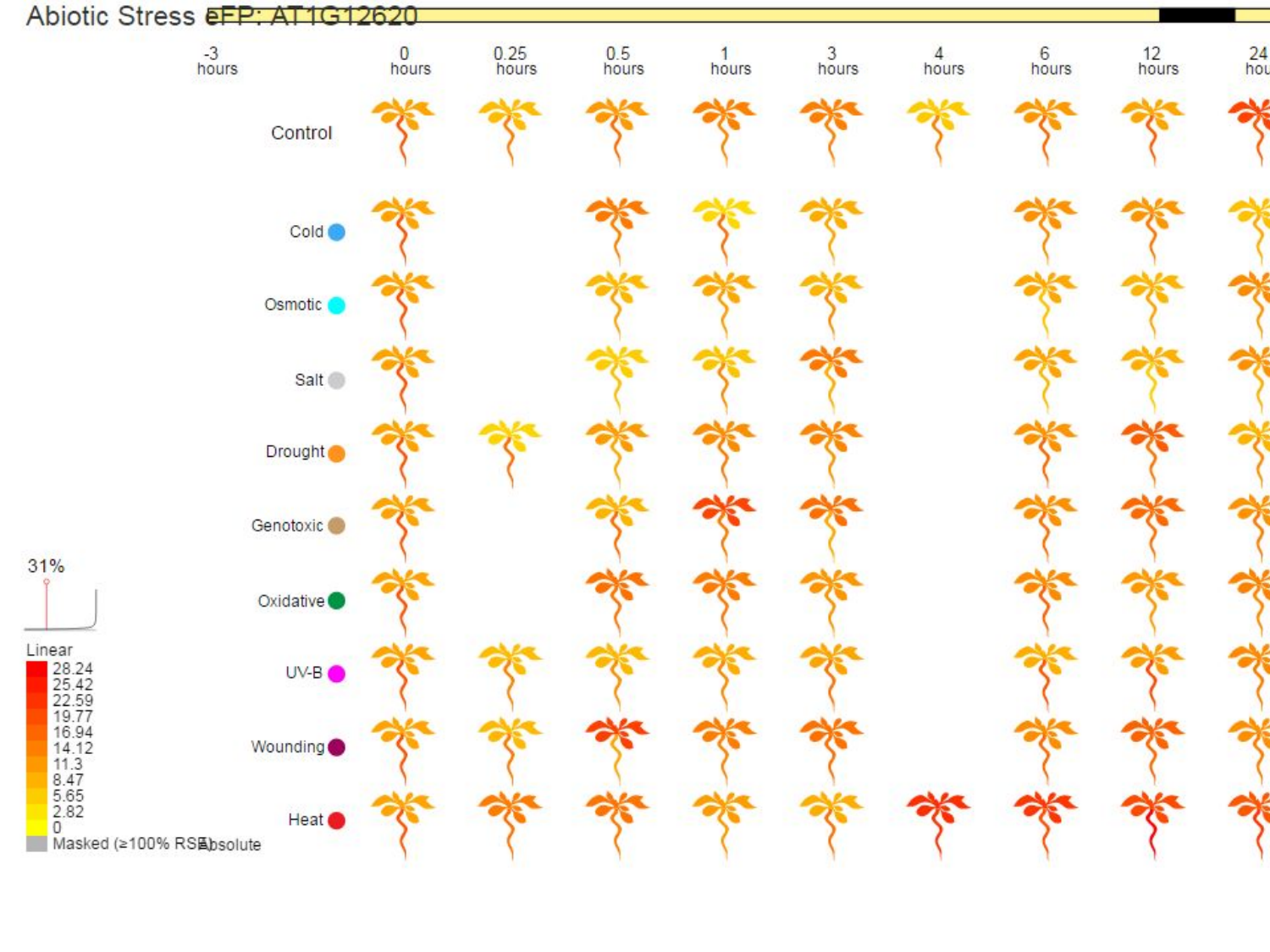

## Slide 16
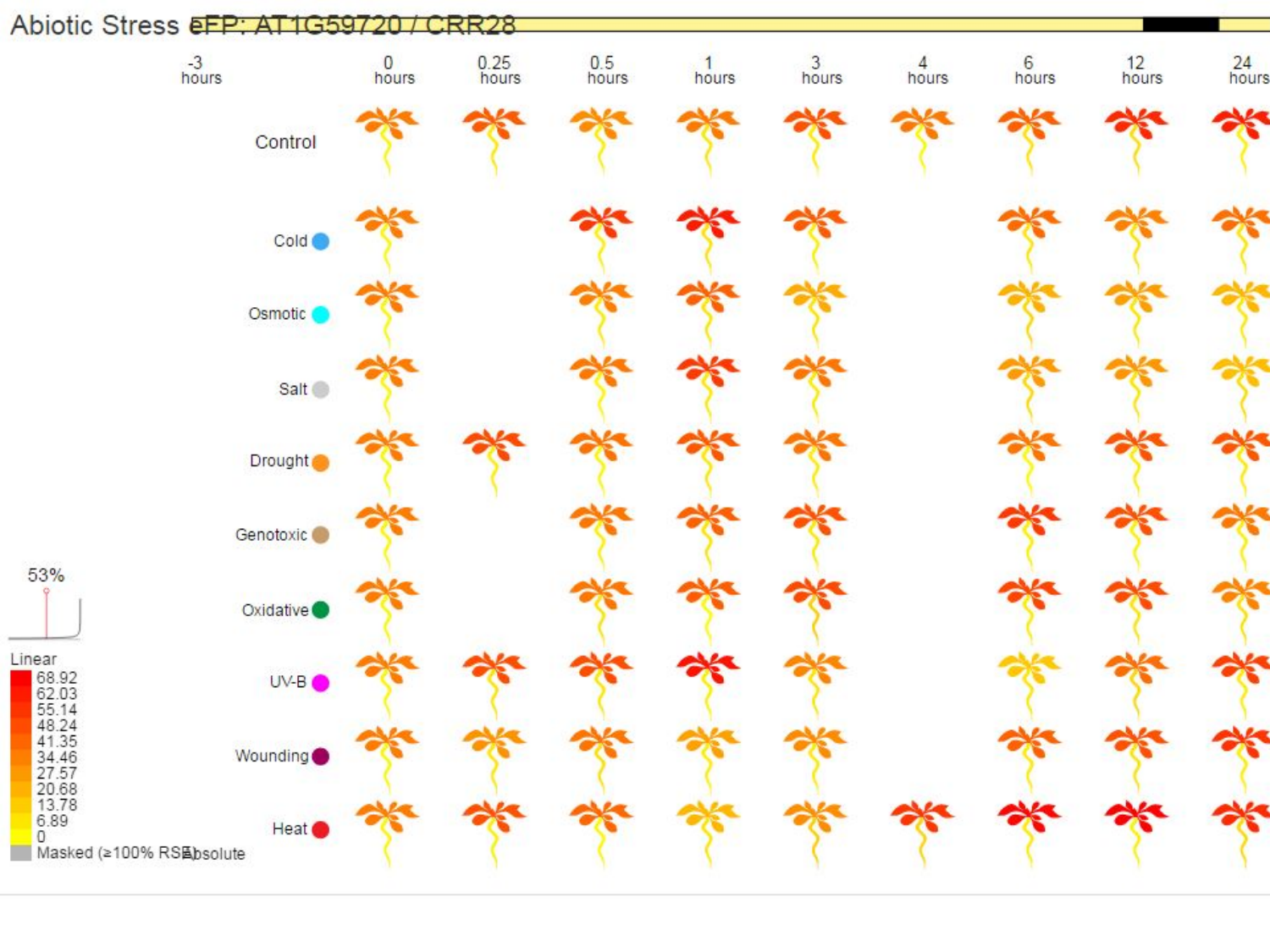

## Slide 17
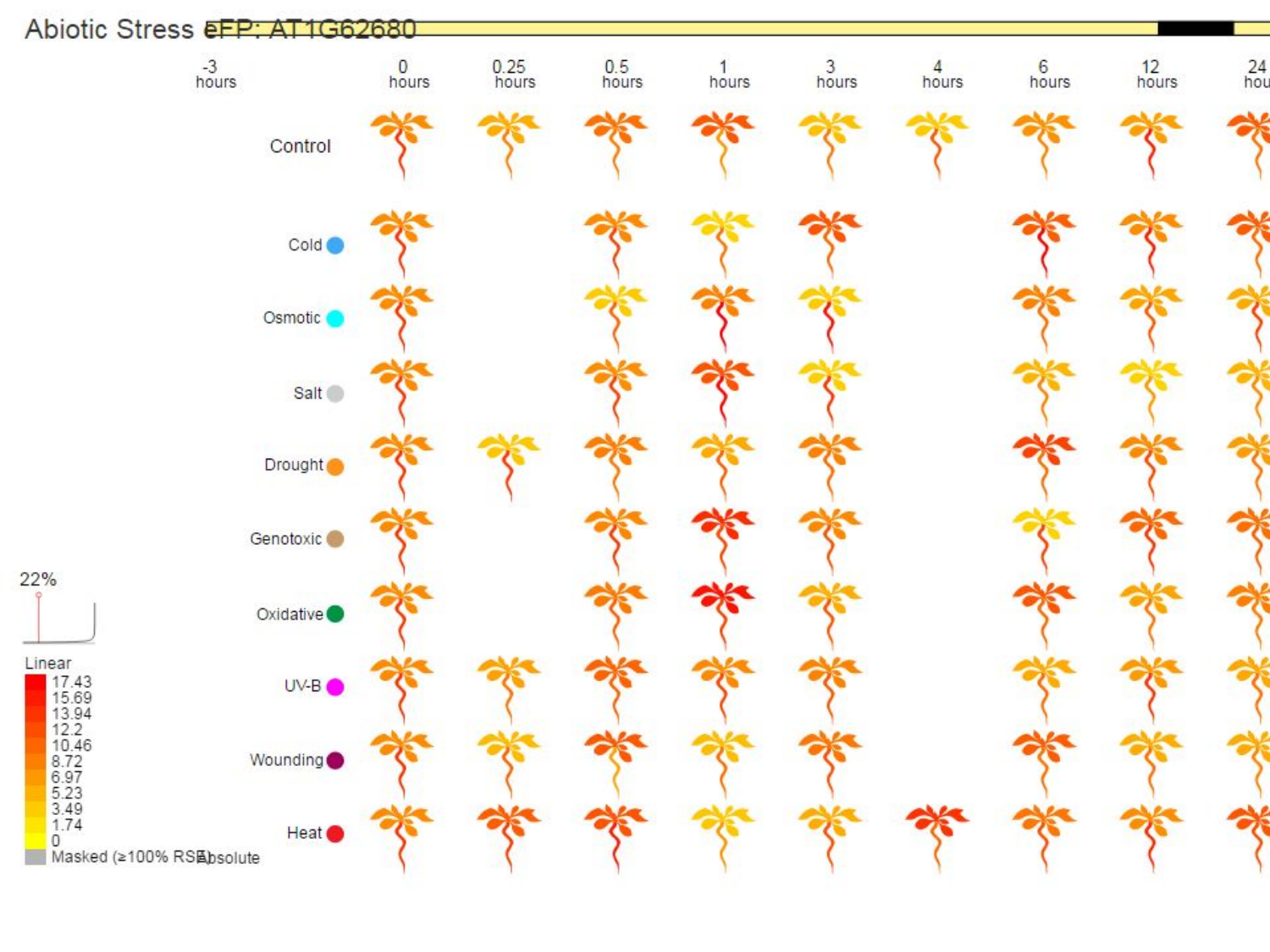

## Slide 18
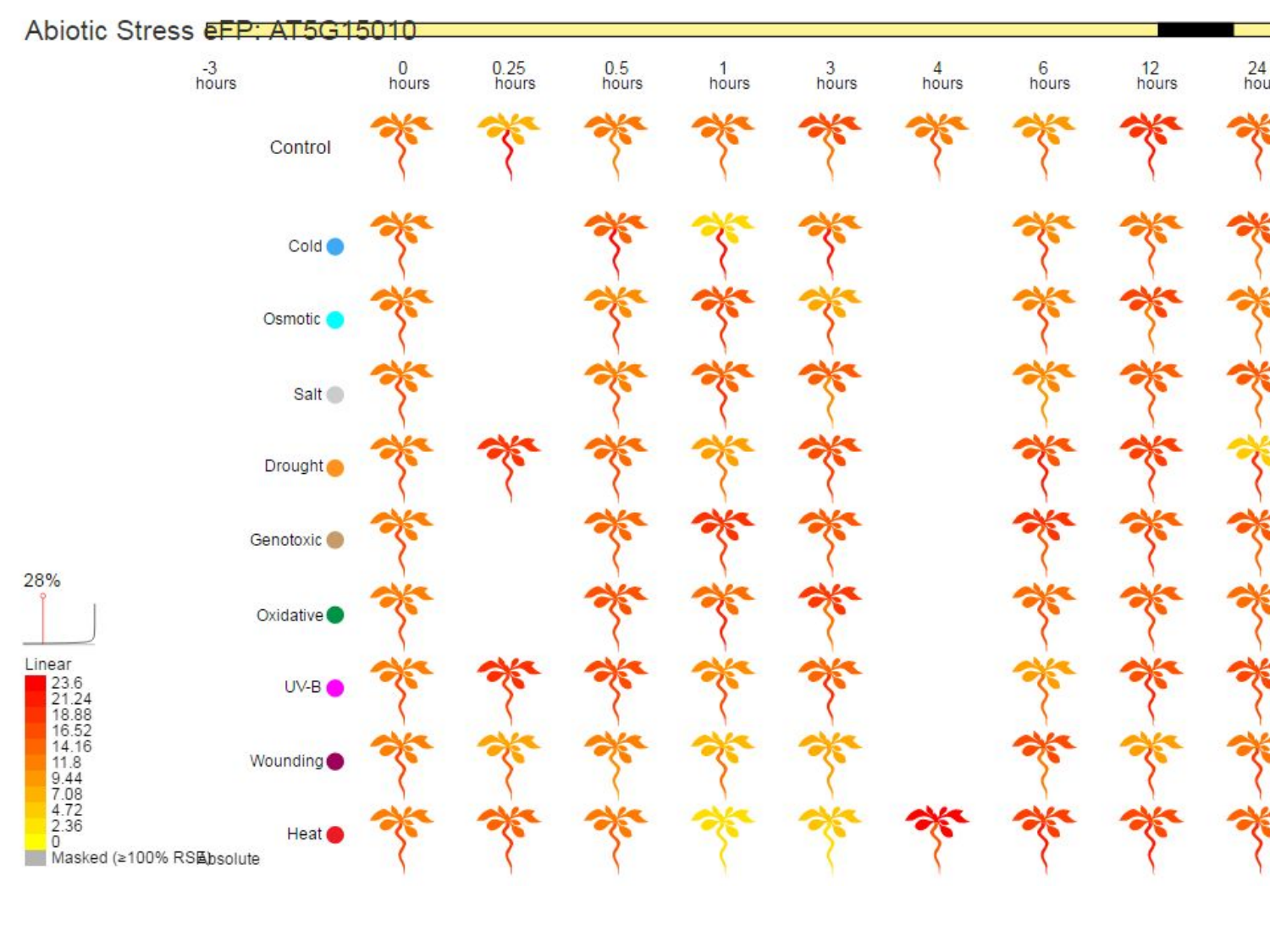

Supplement: Supplementary file 1 [file ijms-25-11065-s001.zip › Figure S2.pptx]
