# Supplementary figures and images for: Rapidly Evolved Genes in Three Reaumuria Transcriptomes and Potential Roles of Pentatricopeptide Repeat Superfamily Proteins in Endangerment of R. trigyna
Source: Int J Mol Sci. 2024 Oct 15;25(20):11065. doi: 10.3390/ijms252011065 (PMC11508020; doi:10.3390/ijms252011065)

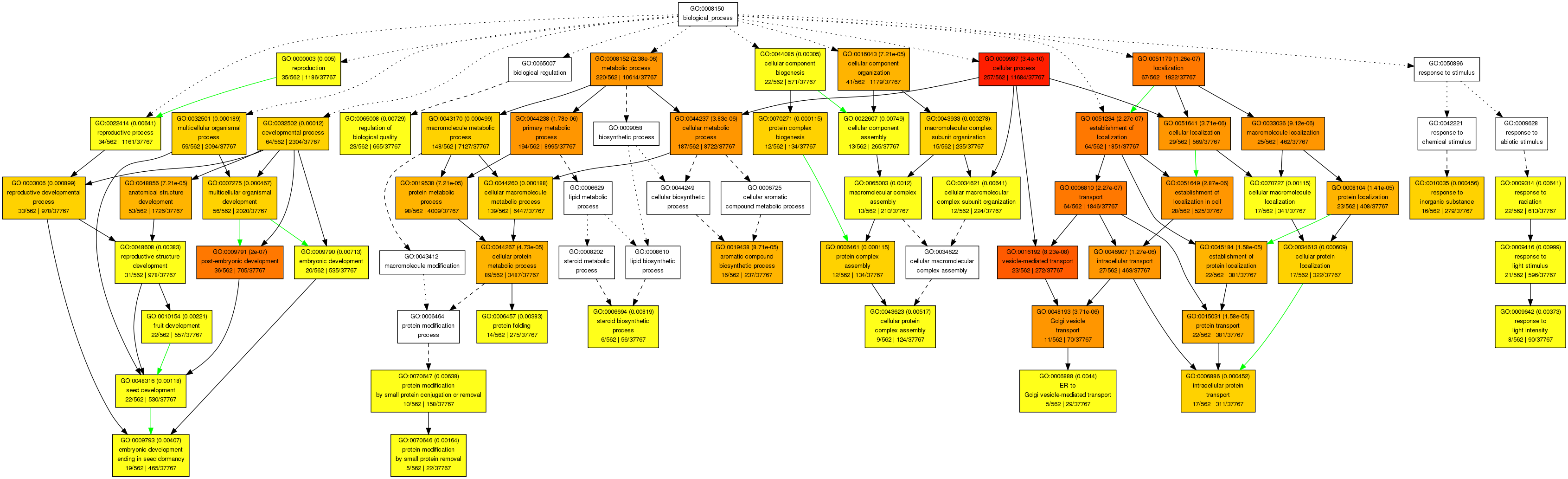

Supplement: Supplementary file 1 [file ijms-25-11065-s001.zip › Figure S1.png]
